# Are molecular methods helpful for the diagnosis of *Helicobacter pylori* infection and for the prediction of its antimicrobial resistance?

**DOI:** 10.3389/fmicb.2022.962063

**Published:** 2022-08-09

**Authors:** Belen Fernandez-Caso, Ana Miqueleiz, Verónica B. Valdez, Teresa Alarcón

**Affiliations:** ^1^Department of Microbiology, Hospital Universitario de Cabueñes, Asturias, Spain; ^2^Department of Microbiology, Hospital Universitario de Navarra, Navarra, Spain; ^3^Department of Microbiology, Instituto de Investigación Sanitaria Princesa, Hospital Universitario La Princesa, Madrid, Spain

**Keywords:** *Helicobacter pylori*, antibiotics, resistance, culture, PCR, GenoType^®^ HelicoDR test

## Abstract

Infections produced by *Helicobacter pylori* (*H. pylori*), a spiral Gram-negative bacterium, can cause chronic gastritis, peptic ulcer, and gastric cancer. Antibiotic therapy is the most effective treatment for *H. pylori* infection at present. However, owing to the increasing antibiotic resistance of *H. pylori* strains, it has become a serious threat to human health. Therefore, the accurate diagnosis of *H. pylori* infections and its antibiotic resistance markers is of great significance. Conventional microbiological diagnosis of *H. pylori* is based on culture; however, successful isolation of *H. pylori* from gastric biopsy specimens is a challenging task affected by several factors and has limitations in terms of the time of response. To improve conventional methods, some molecular techniques, such as PCR, have been recently used in both invasive and non-invasive *H. pylori* diagnosis, enabling simultaneous detection of *H. pylori* and point mutations responsible for frequent antibiotic resistance. The advantages and disadvantages of molecular methods, mainly PCR, versus conventional culture for the *H. pylori* identification and the detection of antibiotic resistance are discussed. As expected, the combination of both diagnostic methods will lead to the most efficient identification of the *H. pylori* strains and the resistance patterns.

## Introduction

*Helicobacter pylori* (*H. pylori*) is one of the most prevalent pathogens worldwide which affects about 50% of the world population (Peleteiro et al., 2014). It colonizes the human stomach, and, although the majority of people (>80%) can remain asymptomatic throughout their life, it is largely related to gastrointestinal diseases. In the absence of treatment, its manifestations can range from pathologies, such as chronic gastritis, peptic ulcers, atrophic gastritis to intestinal metaplasia, gastric cancer, and mucosa-associated lymphoid tissue (Sirous et al., 2011).

The bases of *H. pylori* treatment are antibiotics. The most commonly used are macrolides (clarithromycin or azithromycin), imidazole (metronidazole or tinidazole), amoxicillin, tetracycline, and levofloxacin. Multiple regimens such as triple therapy, sequential therapy, quadruple therapy, and levofloxacin-based triple therapy (Malfertheiner et al., 2017) have been evaluated. Nevertheless, the successful eradication treatment regimen has not yet been achieved due to the increasing rates of resistance to antibiotics included in current regimens. Subsequently, a substantial fall in *H. pylori* treatment efficacy has been observed globally making the eradication of *H. pylori*, a major public health problem (Thung et al., 2016).

Enhanced efforts are required for improving diagnostic tools for *H. pylori*, as well as for a clearer understanding of the development and spread of drug-resistant bacteria. Likewise, the study of antibiotic susceptibility is key to implement the most appropriate treatments and to define treatment guidelines since the choice of antibiotics must be tailored according to local resistance (Malfertheiner et al., 2017).

Clinical tests for *H. pylori* identification that are also useful for the detection of antibiotic susceptibility include culture, as conventional method, and molecular techniques, usually involving gastric biopsy. Although bacteriological culture is considered the reference method for diagnosing infection, *H. pylori* is regarded as a demanding bacterium as it requires supplemented means for its growth, microaerophilic atmosphere, and prolonged incubation which leads to time-consuming limitations when performing sensitivity studies (e.g., Wang et al., 2015). Due to the technical difficulties of *H. pylori* culture, it is shown that easier and faster techniques are required to detect *H. pylori* and to determine its resistance. In this regard, molecular methods have shown remarkable results (Alba et al., 2017).

This article summarizes our current viewpoint of molecular methods, focusing on polymerase chain reaction (PCR) and its applications, both in the diagnosis of *H. pylori* infection and the prediction of its resistance to antibiotics.

## Polymerase chain reaction for *Helicobacter pylori* detection

In recent years, many molecular methods have been developed as alternatives for the identification of *H. pylori*. Most of them are based on PCR or real-time PCR (RT-PCR) directly from gastric biopsies. Other molecular methods that have been tested include nested-PCR, droplet digital PCR, fluorescent *in situ* hybridization, and next-generation sequencing, such as 16S rRNA amplicon sequencing, transcriptomics, and metagenomics (Gong and El-Omar, 2021).

There are several molecular assays commercially available for the identification of *H. pylori*. The extraction of genetic material (DNA) directly from the sample allows for the detection of *H. pylori* through the amplification of specific genes, mainly conserved regions of the *H. pylori* genome. The most used genes are the ureA, ureC, glmM, and Hsp60 or the 16SrRNA or 23S rRNA regions (e.g., Clayton et al., 1991; Wang et al., 2015; Sulo and Šipková, 2021).

The advantage of molecular biology techniques over other methods remains in the increase of sensitivity and the fact that they may allow quantification of the bacteria (Shukla et al., 2011; Belda et al., 2012). However, false-negatives and false-positives arise from the primers employed, due to polymorphism or the use of non-specific primers, respectively (Sulo and Šipková, 2021).

It has been suggested that nested-PCR can achieve sufficient specificity and is much more sensitive than regular PCR as it involves two rounds of amplification, enabling it to amplify the target sequence in a lower concentration (Šeligová et al., 2020; Sulo and Šipková, 2021).

In addition, the combination of several target genes for detection, such as ureA, ureC, glmM, Hsp60, 16S rRNA, 23S rRNA, and vacA, may help to improve the diagnostic performance by reducing the number of false positive results (e.g., Wang et al., 2015; Wongphutorn et al., 2018).

On the other hand, as an alternative to the use of gastric biopsies specimens, different studies that perform PCR on gastric juice (Hsieh et al., 2019) or on non-invasive samples, such as saliva (Sayed et al., 2011) and stool (Beckman et al., 2017; Leonardi et al., 2020), have been published obtaining promising results. So far, there are different sensitivities and specificities depending on the DNA extraction method and the PCR assay used. Other limitations found are requirements of facilities and experts, smaller abundance of the bacteria, presence of an inhibitory substance, and interference from the dead bacteria or DNA degradation.

## Polymerase chain reaction for detection of resistance

Molecular techniques also allow the detection of antibiotic resistance. *H. pylori* strains have developed different mechanisms of antibiotic resistance, such as point mutations or other genetic changes. Molecular mechanisms underlying this resistance have been intensively studied, especially in the cases of clarithromycin and levofloxacin, the most commonly used antibiotics for *H. pylori* eradication, given that the vast majority of resistance to these two antibiotics is due to known localized mutations.

Hence, by targeting the genes responsible for antibiotic resistance, it is feasible to obtain genotypic susceptibility information for the common antibiotics used, without performing an antibiogram. Furthermore, the detection of genes and mutations involved in antibiotic resistance using molecular techniques has been already acknowledged as a useful tool to be performed directly on the gastric biopsy specimen by Maastricht V/Florence Consensus Report (Malfertheiner et al., 2017).

Polymerase chain reaction is the most widely used molecular determination approach (Hortelano et al., 2021). Several commercial kits (Cambau et al., 2009; Nishizawa and Suzuki, 2014; Redondo et al., 2018) are available to detect different mutations that confer resistance to clarithromycin combined or not with levofloxacin. PCR has also been applied to detect resistance to tetracycline and rifampicin by in-house protocols which are largely described in the literature (Chisholm and Owen, 2009; Contreras et al., 2019).

When referring to clarithromycin, most methods identify the main mutations which are found in the 23S rRNA gene, targeting point mutations at nucleotide positions A2146 and A2147 (Redondo et al., 2018). These two mutations were previously named 2,142 and 2,143 (Wang and Taylor, 1998). For its part, resistance to fluoroquinolones is due, in a very significant percentage, to mutations in genes gyrA and gyrB DNA gyrase, positions 86, 87, 88, and 91 (Rimbara et al., 2012). Also, PCR techniques have been applied to detect tetracycline resistance (Contreras et al., 2019) due to mutations in the 16S rRNA gene (positions 926–928) and rifampicin in the rpoB gene (positions 525–545 and 547) (Chisholm and Owen, 2009). In the case of metronidazole, its resistance mechanisms are quite complex (Nishizawa and Suzuki, 2014) and not as well defined, so for the moment, there are no commercial kits for its detection.

## Polymerase chain reaction *vs.* culture

Keeping the above observation in mind, we have compared *H. pylori* identification performance efficacy among culture and molecular *H. pylori* identification GenoType^®^ HelicoDR kit (HAIN Life Science, Hardwiesenstraße, Germany). In terms of antibiotic resistance, phenotypic susceptibility was studied in all culture-positive and was compared to GenoType^®^ HelicoDR kit which allows simultaneous detection of *H. pylori* and its resistances to clarithromycin and levofloxacin and is applicable to gastric biopsy specimens. The Genotype^®^ HelicoDR test allows the detection of point mutations responsible for the resistance to clarithromycin and levofloxacin: three-point mutations in the V domain of the 23S rRNA gene (positions 2,146 and 2,147) for clarithromycin and 4 mutations in the A subunit of DNA gyrase (1 at codon 87 and 3 at codon 91) for quinolones.

### Patients and methods

The specimens used for this study were gastric biopsy samples taken from 616 patients aged between 2 and 82 years old who had undergone endoscopy due to gastroduodenal diseases. One sample per patient was analyzed. Data including age and sex were collected. The study was performed from January 2016 to October 2017 and samples were collected from a variety of Spanish hospitals. Gastric biopsy specimens of the patients were referred to the Clinical Microbiology laboratory of Hospital Universitario La Princesa in Madrid. Freshly taken biopsy specimens were either placed into Portagerm Pylori (BioMérieux) solution and sent within 24 h under room temperature or into sterile glass tubes and kept at 4°C. Once at the laboratory, if the process could not be continued within 3 h, the samples were stored at −80°C deep freezer until further processing.

Biopsy specimens’ culture was performed in a biological safety cabin and then preserved at −80°C deep freezer for further molecular processing. The specimens were discharged with the aid of a sterile swab into two commercial culture media: blood agar (Columbia agar + 5% sheep blood, BioMérieux) and Helicobacter selective agar media (Pylori agar, BioMérieux). The samples were incubated for 15 days at 37°C in a micro-aerobic atmosphere (5% O_2_, 10% CO_2_, and 85% N_2_). *H. pylori* colonies are small, translucent to yellowish colonies, which can be identified based on a Gram-negative helical-shaped in Gram-staining procedure followed by positive oxidase, catalase, and urease tests, along with the confirmation by MALDI-TOF mass spectrometry (Bruker).

The antibiotic sensitivity study was performed using the gradient strip diffusion method in blood agar plates. *H. pylori* isolates collected were examined for their susceptibility to antimicrobial agents by gradient diffusion strip method (*E*-test, BioMérieux) on blood agar incubated at 37°C in a micro-aerobic atmosphere (5% O_2_, 10% CO_2_, and 85% N_2_). Six antimicrobial agents were tested: clarithromycin, metronidazole, amoxicillin, levofloxacin, tetracycline, and rifampicin, on which MIC values were read after 3 and 5 days and interpreted according to the clinical breakpoints of EUCAST guidelines.

For the molecular method, DNA extraction included a previous stage meant for the digestion of proteins. Solid biopsies were mixed with Proteinase K, lysis buffer, and distilled water followed by incubation in agitation for at least 3 h at 37°C. The DNA isolation was carried out by automated DNA extraction using the NucliSens^®^ easyMAG™ (BioMérieux), following the manufacturer’s description for the tissues. All the eluted DNA were stored at −80°C.

The biopsy material itself, as well as the culture material extracted from it, can be used as the starting material. Briefly, multiplex amplification of DNA regions of interest was performed. Typical PCR reaction mixtures contained 5 μl of reaction buffer, 2.5 μl of MgCl_2_, 35 μl of 5′-biotinylated primers and nucleotide mixture, 0.4 μl of Taq polymerase, 2.5 μl of PCR grade water, and 5 μl of extracted DNA. The PCR run comprised 30 cycles. The denaturation cycle was 1 cycle at 94°C for 5 min. Then, 30 cycles which were composed of a first step at 94°C for 30 s, a second step at 55°C for 30 s, and a third step at 72°C for 30 s. The PCR ended with 7 min at 72°C. The test was developed and interpreted according to the manufacturers’ instructions.

The PCR was followed by hybridization with DNA strips, coated with different specific oligonucleotides (probes). Hybridization was performed using the TwinCubator (Hain Life Science) system at a temperature of 45°C. The denaturation solution was mixed with 20 μl of the amplified sample and was hybridized using a standard hybridization protocol. Each strip contains a total of 18 hybridization probes. The first band contains the conjugate control designed to indicate effective binding to the substrate. The second band includes a universal control, and it is used to check that the amplification has taken place correctly. The third band contains a sequence from a region of the 23S rRNA that is common to all *H. pylori* strains. The next ten and five bands are for quinolone and clarithromycin sensitivity studies, respectively. The probes were designed to hybridize with both the sequences of the wild-type (WT) and the mutated alleles (MUT).

Interpretation was performed after strips were attached to the evaluation sheet after hybridization, with the template aligned side by side with the conjugate control band of the respective strip. Control bands that should appear positive to validate the test. A positive band was determined by comparing each band stain with the amplification control band. A stronger stain than the amplification band was interpreted as positive for the presence of the allele. WT specimens were determined by the presence of WT bands only. *H. pylori* strain mutations included clarithromycin resistant, fluoroquinolone resistant, and resistant to both antibiotics. Mutants were determined by the presence of the MUT band.

## Results

Therefore, a total of 616 specimens were examined for *H. pylori* presence using the two methods. Results indicate that 234 (37.9%) were found positive for the presence of the *H. pylori* bacterium by both methods and 308 (50%) were negative. In terms of discrepancies observed, 2 (0.32%) specimens were only detected by culture. On the contrary, 72 (11.6%) specimens were found positive only by means of PCR. If we consider culture as gold standard, the comparison revealed a sensitivity of 99.1% and a specificity of 81% in favor of the molecular kit. It should be borne in mind that the positives by PCR and negative by the culture were not false positives, as the PCR was able to detect more positives than the culture.

Regarding the *E*-test susceptibility testing, it was not possible to determine antibiotic sensitivity in the 236 isolates for all antibiotics; only 223 isolates could be studied for clarithromycin and levofloxacin. For clarithromycin, 107 (47.9%) isolates had a sensitive phenotype; while for levofloxacin, 206 (92.3%) isolates had a sensitive phenotype. Despite the isolation of H. pylori in culture from 236 strains, the antibiogram was not viable in all of them. Only the sensitivity of 223 strains could be studied phenotypically while in 13 strains, the antibiogram was not viable.

Resistance to clarithromycin was 52% for fenotypic while 59% for genotypic method and to levofloxacin was 76% for fenotypic while 84% for genotypic method (No statistically significant differences).

On the other hand, the results of the genotypic study of antibiotic sensitivity to clarithromycin and levofloxacin performed with GenoType^®^ HelicoDR are shown in Table 1.

**TABLE 1:** Genotypic determination of clarithromycin and levofloxacin resistance mutations by PCR.

Antibiotic			n (%)
**Clarithromycin**	No mutation		123 (40.2)
	Detected mutation	A2146G	12 (3.9)
		A2146C	2 (0.7)
		A2147G	165 (53.9)
		Double mutation	4 (1.3)
	**Total**		306 (100)
**Levofloxacin**	No mutation		261 (91.6)
	Detected mutation	N87K	9 (3.1)
		D91N	5 (1.7)
		D91G	6 (2.1)
		D91Y	4 (1.4)
	**Total**		285* (100)

*In 21 samples the PCR result for levofloxacin resistance was indeterminate.

When comparing the results obtained in all samples in which both phenotypic antibiotic sensitivity and genotypic PCR were available, a difference between the two antibiotics was observed (Table 2).

**TABLE 2:** Presence or absence of PCR mutations for clarithromycin and levofloxacin versus sensitivity or resistance results by *E*-test (gold standard).

		Susceptible by *E*-test	Resistant by *E*-test	Total	Sensitivity	Specificity
Clarithromycin PCR	Mutation	21 (15.4%)	115 (84.5%)	136	99.1%	80.0%
	No mutation	84 (98.8%)	1 (1.1%)	85		
Levofloxacin PCR	Mutation	0 (0%)	16 (100%)	16	100%	100%
	No mutation	198 (100%)	0 (0%)	198		

Based on these results, a sensitivity of 99.1% and a specificity of 80% were calculated for the detection of clarithromycin resistance by the genotypic PCR method. For levofloxacin, no discrepancy was observed between the two methods, so the sensitivity and specificity for the detection of levofloxacin resistance by PCR were 100%.

## Discussion

Efficient diagnosis is required for successful clinical management to relieve symptoms and to eradicate bacteria. As with any diagnostic method, culture and molecular techniques have advantages, but also disadvantages.

Molecular techniques for the diagnosis of *H. pylori* have a very high sensitivity (over 95%), detecting more positive specimens than culture, and specificity (close to 100%). PCR techniques do not depend on the lability of the bacteria, as culture does. In addition, some molecular methods can be applied directly on the sample, gastric biopsy or non-invasive samples. Moreover, in relation to working time, amplification of genome specific regions in *H. pylori* using appropriate primers allows faster identification than culture, as *H. pylori* takes 10–14 days for cultures to be negative.

The advantages of genotypic methods over phenotypic methods include providing insight into underlying resistance mechanisms in a fairly short time (<4 h).

Meanwhile, among the disadvantages, are the higher price in comparison to conventional methods and the need for appropriate equipment and experience personnel. That is the main reason why approaches involving DNA amplification have not been widely accepted in general practice.

It is worth mentioning, as an alternative to the use of gastric biopsies, the appearance of PCRs for the diagnosis of *H. pylori* directly from non-invasive samples. This could be a great advance as it avoids the need for endoscopy and biopsy, thus preventing all the discomfort and risks that they entail for the patient, as well as the associated healthcare costs.

Besides, molecular techniques have limitations when it comes to detecting antibiotic resistance. The main disadvantage of PCR in detecting resistance as opposed to *H. pylori* culture is that the most commercial systems only detect specific resistance mutations to clarithromycin and quinolones, whereas culture allows the study of sensitivity to all antibiotics and the detection of resistant isolates by other mutations or resistance mechanisms. They only detect specific mutations, so there is a possibility of not detecting resistant isolates due to mutations other than those amplified by PCR or resistant isolates due to other resistance mechanisms unrelated to these.

In conclusion, both methods are still necessary for the study of sensitivity, and neither should be displaced. These procedures could represent an essential component of the efforts needed to prevent the further development of infections caused by *H. pylori* and the spread of the increasing resistance to antibiotics.

## Data availability statement

The original contributions presented in this study are included in the article/supplementary material, further inquiries can be directed to the corresponding author.

## Ethics statement

Ethical review and approval was not required for the study on human participants in accordance with the local legislation and institutional requirements. Written informed consent for participation was not required for this study in accordance with the national legislation and the institutional requirements.

## Author contributions

TA conceived the presented idea and was in charge of overall direction and planning. VV carried out the experiments. AM contributed to the interpretation of the results. AM and BF-C wrote the main manuscript text. TA, AM, BF-C, and VV contributed to the final manuscript. All authors contributed to the article and approved the submitted version.
